# 

**Published:** 1916-11

**Authors:** 


### Contents

Event and Comment .......... 501

Supplee Impression Method .......... 504

A Proper Dietary for Dentists .......... 516

Personal Experience .......... 521

Mouth Infections .......... 522

Announcements .......... 528

Bibliography .......... 539

Obituary .......... 542

